# Comparing attitudes towards compulsory interventions in severe and persistent mental illness among psychiatrists in India and Switzerland

**DOI:** 10.1186/s12888-024-05710-6

**Published:** 2024-04-18

**Authors:** Christina Rickli, Julia Stoll, Anna Lisa Westermair, Manuel Trachsel

**Affiliations:** 1 Institute of Biomedical Ethics and History of Medicine, University of Zurich (UZH), Zurich, Switzerland; 2 Clinical Ethics Unit, University Hospital Basel (USB) and University Psychiatric Clinics (UPK) Basel, Basel, Switzerland; 3 Faculty of Medicine, University of Basel, Basel, Switzerland

**Keywords:** Psychiatrists, Coercion, India, Switzerland, Schizophrenia, Depression, Autonomy

## Abstract

**Background:**

Psychiatrists face a major ethical challenge when deciding whether to make use of coercive measures in the treatment process of patients suffering from severe and persistent mental illness (SPMI). As India and Switzerland show major cultural, political and financial differences, it is hypothesized that attitudes towards coercive measures among Indian and Swiss psychiatrists will vary too. Exploring differences in attitudes between cultures strengthens the critical reflection on one’s own stances and in consequence, on our way of action. Especially when it comes to situations involving power imbalances between patients and health practitioners, self-reflection is essential to prevent ethically inappropriate behavior.

**Methods:**

An online survey on aspects of care for patients with SPMI was sent to 3’056 members of the Indian Psychiatric Society between April and June 2020 and to 1’311 members of the Swiss Society for Psychiatry and Psychotherapy between February and March 2016. The respondents’ answers were compared. This article deals with the questionnaire’s items on autonomous decision making and the implementation of coercive measures in clinical practice. More precisely, participating psychiatrists were asked to rate the importance of patient’s autonomy in general and their willingness to apply coercive measures regarding two specific case vignettes depicting a patient with schizophrenia and one with depression. The statistical analysis, namely descriptive data analysis and calculation of arithmetic means, Shapiro Wilks tests and Mann-Whitney *U* tests, was carried out using IBM SPSS Statistics version 27.

**Results:**

Answers were received from 206 psychiatrists in India and 457 psychiatrists in Switzerland. Indian participants tended to value autonomous decision making as slightly less important than Swiss participants (62.2% vs. 91%, *p* =.01). Regarding a case of severe and persistent depression, psychiatrists in the Indian group were on average more in favor of acting against the wishes of the patient (55% vs. 34.1%, *p* <.0001) as well as of accepting a temporary decrease in quality of life due to coercion (40% vs. 23%, *p* =.008). Answers concerning a case of schizophrenia revealed that Indian participants were more in favor of acting against the patient’s wishes than Swiss participants (39% vs. 37%, *p* =.007), whereas the comparison whether to accept a temporary decrease in quality of life regarding this case showed no significant difference (*p* =.328).

**Conclusions:**

The significant difference in attitudes towards coercive measures among Indian compared to Swiss psychiatrists found in this study might arise from a predominantly more collectivist society in India compared to Switzerland. Moreover, differences in financial resources, the organization of the health care system, and the historical background might have an influence. Continuous and critical reflection on one’s own views and behavior is essential, especially if ethical principles and individual rights could be violated through a power imbalance, as in the case of coercive measures.

**Supplementary Information:**

The online version contains supplementary material available at 10.1186/s12888-024-05710-6.

## Background

Providing adequate treatment for patients suffering from *severe and persistent mental illness* (SPMI; for definitions see [1, 2]) is a major challenge in psychiatric practice. Frequent and long-lasting hospitalizations, psychosis or violent behavior due to their illness might make them prone to *compulsory interventions* [3] defined as measures contrary to the person’s will or applied against the patient’s non-verbal or verbal resistance [4]. Coercion can be applied in different ways, including involuntary admission or treatment, seclusion in a locked room or mechanical as well as physical restraint. Also, verbal, and nonverbal methods of exerting pressure to influence or control another person can be counted as coercion. Those so called informal coercive methods include persuasions, interpersonal leverage, inducements, or threats [5]. In Switzerland, up to 11% of all psychiatric inpatients [5, 6] and 28% of the involuntary admitted patients [3] experience at least one coercive measure. These numbers are in line with the data from involuntary admitted patients in 10 European countries, revealing a prevalence of coercive measures between 21 and 59% with a high variability between countries [7]. In India, data collected in a government hospital in Bengaluru showed that 66% of the inpatients experienced coercion [8]. To the authors knowledge, there is no existing nation-wide data on the prevalence of coercive measures in psychiatry all over India. The prevailing coercive method in Switzerland is seclusion followed by the use of chemical restraint [6]. In contrast, in India seclusion is prohibited by law and the data collection from the previously mentioned hospital in Bengaluru showed chemical or physical restraint to be the most applied coercive measures [8]. Another frequently applied coercive measure in India is putting covert medication in drinks or food of mentally ill patients, referred to as surreptitious treatment. For a study conducted in Chennai, family members of patients suffering from schizophrenia were interviewed regarding surreptitious treatment methods. It revealed that 50% of non-compliant patients have received hidden medication by their family members [9]. There was no data found on the prevalence of covert/concealed treatment methods in Switzerland.

The use of coercive measures highly interferes with the autonomy of the affected individuals. But what is meant by autonomy? In the following paragraph, different aspects of autonomy are presented to provide a deeper insight into the topic.

Autonomy is a very broad concept which makes it unwieldy when it comes to making ethically sound decisions in a relatively short period of time and thus for everyday use. Nevertheless, it is essential to keep the interdisciplinary dialogue between philosophy and psychiatry going; without its empirical scope, autonomy would remain an empty and abstract concept [10]. One way to discuss different aspects of autonomy is by drawing on Isaiah Berlin’s “two concepts of liberty”, where he describes a positive and a negative sense of freedom [11].

Autonomy can represent freedom of action, also referred to as positive self-determination or positive freedom [12]. By asking participants about the importance of autonomous decision making of SPMI patients regarding the treatment process, we most likely picture this aspect of autonomy. Our survey addressed the patient’s possibility to decide on the next steps, whether to receive treatment and how it should look like. In general, this first aspect of autonomy contains the freedom of choice on pretty much every decision we make, and ranges from deciding what kind of ice cream to have for dessert to where we want to life or whom we want to date [13]. Further, autonomy includes the right of non-interference (negative freedom) [12], in which the right to physical integrity is founded. Our survey considered this second aspect through the questions regarding the case vignettes: “In this case I would accept a temporary decrease in quality of life due to coercive measures” and “In this case, I would not proceed against the patient’s wish”. The two so far discussed aspects of autonomy are both conflicted when coercive measures are applied in mental health care.

As a further aspect, autonomy also is said to include self-purposefulness or dignity describing the value of a person that arises from its mere existence, cannot be compared among individuals, and is not bound to its functioning [12]. This goes back to Immanuel Kant, who stated that humanity should never be treated merely as a means, but always as an end in itself [14]. In the course of mental illness, psychopathological phenomena might impair a person’s functioning and power of judgement. Situations may occur when coercive measures present beneficial and justified. However, violating a person’s dignity is never justified and as applying a coercive measure creates an exceptional situation in which the dignity of the affected person becomes very tenuous, it should be psychiatry’s goal to protect it. It does not end with the decision whether to apply coercion or not, as the focus should be on how coercive measures are implemented. Essential is a well-established therapeutic relationship [15–17], which from the viewpoint of the ethics of care is based on mutual respect and perceiving individuals as fundamentally autonomous human beings tightly integrated in and influenced by their relationships [10].

As coercive measures jeopardize the fundamental rights of every human being such as the right to freedom, self-determination, and physical integrity [18], they are frequent subjects to controversial debates. Consequently, laws have been set up to provide more clarity for their application in clinical practice. The legal basis for coercive measures in Switzerland and India is similar. In both countries, the use of coercion is limited to settings where no less constringent alternative is applicable to ensure the safety of the concerned patient or third parties. If a coercive method is applied, the least restrictive alternative must be chosen, its adequacy must be reviewed regularly, and the measure must be stopped immediately as soon as it proofs unnecessary (Switzerland: ZGB 426–439; India: MHCA2017 Chapter XII). From an ethical point of view, using coercion should only be considered if the patient is lacking decision making capacity [19]. They form exceptional measures targeted to restore or maintain the patients’ health [4], and aim for a fast symptom reduction which will enable the patient to make his/her own decisions again [18].

Despite those regulations, recognizing whether any benefit results from applying coercive measures poses a major challenge to psychiatrists. The four principles of biomedical ethics by Beauchamp and Childress– respect for autonomy, justice, non-maleficence and beneficence– are useful to depict the underlying problem in a specific case [20]. For example, when psychiatrists apply coercion, they aim to achieve beneficence for the patient, but inevitably come into conflict with the principles of respect for autonomy and non-maleficence [21]. However, when applied to clinical decision making the model’s practical implications are limited since it provides no step by step approach on how to proceed when weighing the principles against each other to arrive at an ethically sound decision. For this, it can be helpful to start from a specific scenario and use guidelines as an orientation to come to a valid solution [12]. Eventually, the process of decision making will be highly influenced by the attitudes of the psychiatrist and thus their personal and societal background. The influence of the societal background on the attitudes towards coercion is subject to this study.

Although both modern democracies, India and Switzerland show substantial differences with regard to their predominant culture, their organization of social structures, and regarding their economic situation and health resources. The Indian society is frequently described as collectivist with people tending to be tightly integrated in groups and common goals being prioritized over individual goals. Hofstede established a measurement to determine a countries’ position on an Individualism-Collectivism Scale and found that individualism prevails in Western countries whereas Asian countries show a greater degree of collectivism [22]. While labelling countries in that way might encourage stereotyping of culturally diverse groups [23], families are undoubtedly playing an important role in the provision of India’s health care [24–27]. In India, it is mandatory for inpatients to be accompanied by a family member while staying at the hospital [24] which can at least partly be explained by the structural organization of India’s health care system and relates to the problem of scarce resources. The presence of a family member during a hospitalization might strengthen a family-centered attitude in India. In addition to the mere presence and support coming from family members in India, literature suggests medical paternalism to be more accepted than in many Western countries [28, 29]. With India’s ratification of the United Nations Convention on the Rights of Persons with Disabilities in 2007 and the subsequent amendments through the Mental Health Care Act 2017 (MHCA2017), a stronger focus is put on the patients’ individual rights and autonomous decision making. Subsequently, concerns have been raised expressing its potential harm for the Indian family-centered health system [30, 31]. Cultural differences between the two countries make a comparison of attitudes towards coercion particularly interesting.

Regarding the economic situation in the two countries, a comparison shows that the gross domestic product per inhabitant amounts 1900 USD in India and 86’000 USD in Switzerland. Its percentage spent on health in 2018 was 3.54% in India and 11.88% in Switzerland while the world average health expenditure was at 9.85% [32]. The density of practicing psychiatrists is approximately hundred times higher in Switzerland compared to India [33]. These numbers illustrate the considerable difference in health resources in the two compared countries. According to caregivers and psychiatrists surveyed at the Krishna Rajendra Hospital in Mysore, India, coercive measures were mainly applied because of scarce resources [34]. Thus, the potential influence due to differing health resources on psychiatrists’ attitudes towards coercion should be considered.

Except for a questionnaire analysis on attitudes towards coercion among psychiatrists in England and Germany, which found similar attitudes in both countries [35], intercultural comparisons on the topic are still missing [36]. By exploring factors which may influence our understanding of coercive measures, we addressed this existing gap. For this study, Indian psychiatrists have been surveyed on their attitudes towards coercive measures and their answers were compared to previously analyzed answers from psychiatrists in Switzerland [37]. Due to the countries’ differences in the prevalence and prevailing method of coercion, its divergent culture and societal system as well as distinct financial resources, the participants’ attitudes towards coercive measures are hypothesized to show significant differences too. Learning about attitudes of psychiatrists in other parts of the world might trigger a deeper reflection on own values and beliefs and might help to set up adequate guidelines for ethical decision making.

## Methods

### Questionnaire and sample

The design and validation of the used questionnaire is described in Trachsel et al. [38] and its revision and adaptations as used in the present study are detailed in Stoll et al. [39]. We used a previously established questionnaire with 23 items on a 7-point Likert scale to assess attitudes towards different goals of care and approaches to care in SPMI. Five items asked about the importance of different aspects in the treatment of patients with SPMI in general, eight items related to palliative psychiatry and SPMI and the remaining items focused on two case vignettes of severe, chronic, and treatment-refractory schizophrenia and major depression. The case vignettes have been adapted and reused from two previous publications [40, 41]. They represent two stories of patients who have undergone various treatment options over several years and still suffer from severe symptoms and a substantial reduction of life quality. Additionally, participants were asked on their age, gender, and year of graduation from medical school (see supplementary materials). For recruitment in India, an email with a standard text and a survey link was sent to 3’056 members of the Indian Psychiatric Society between April and June 2020. Participants in Switzerland were recruited from February to March 2016 by contacting all German-speaking members of the Swiss Society for Psychiatry and Psychotherapy (SSPP, 1311 members). Furthermore, participants have been informed that there was no advantage or disadvantage due to participation, and answers were saved anonymously. All participants gave their informed consent to participate. The online survey was established using the SoSci Survey tool (SoSci Survey GmbH) and answers from the group in India were compared to the answers from the group in Switzerland. Here, we report results relating to the use of coercion from India and compare them to the previously published results from Switzerland [37], specifically the item “In the treatment of patients with SPMI, how important is the patient retaining decision making autonomy?” and the two items concerning the case vignettes “In this case, I would not proceed against the patient’s wishes” and “In this case, I would accept a temporary decrease in quality of life as a consequence of coercive measures.”

### Statistical analysis

Data was analyzed using IBM SPSS Statistics® version 27 (IBM Corp. Released 2020. IBM SPSS Statistics for Macintosh, Version 27.0. Armonk, NY: IBM Corp). To improve readability, the 7-point Likert scales were collapsed into the three categories *unimportant* [1–3], *moderately important* [4] and *very important* [5–7] for the question regarding autonomous decision making, and *disagreement* (-3/-2/-1), *neutrality* (0) and *agreement* [1–3] for the questions in relation to the case vignettes. As the data significantly deviated from normal distribution in the Shapiro Wilks test, we used the Mann-Whitney *U* test for inter-country comparison. To interpret the importance of a calculated difference in attitudes and to make results comparable to other research, we calculated the commonly used correlation coefficient *r* as a measure for effect size [42]. Cohen’s suggestions for interpretation of *r* with *r* <.3 denoting a small, 0.3 ≤ *r* <.5 a medium, and 0.5 ≤ *r* a large effect were used [43].

Partial correlations were calculated to check for a potential influence of the participants’ age and their time since graduation on their answers. To assess the influence of a diverging response style pattern between the two groups, frequencies of choosing extreme or neutral answers on the 7-point Likert scale were summed up per group. The response style pattern were compared using a t-test and the corresponding effect size was stated by calculation of Cohen’s *d*.

## Results

Through the recruitment process, answers from overall 206 psychiatrists in India (response rate 6.7%) and 457 psychiatrists in Switzerland (response rate 34.9%) were collected.

### Sample characteristics

The sample in India consisted of 67% male and 33% female participants. This gender distribution did not differ significantly from the gender distribution in the sample in Switzerland (*U* = 47629.00, *p* =.172 and *r* =.05). Participants in India showed an average age of 43.1 years (*Mdn* = 40, range = 25–78 years) and a mean time since graduation of 19 years (*Mdn* = 15.5, range = 2–56 years). Years from graduation and the participants’ age differed from normal distribution with a tendency towards young and close to graduation participants. Both, age and time since graduation were significantly different in the sample in India compared to the sample in Switzerland (with *U* = 73876.50, *p* <.0001 and *r* =.52 regarding the age and *U* = 65422.00, *p* <.0001 and *r* =.38 regarding the time since graduation). Participants in Switzerland tended to be older than participants in India with an average age of 57.7 years (*Mdn* = 58, range = 35–88 years) and further from graduation with a mean time since graduation of 27 years (*Mdn* = 27.0, range 8–65 years).

Considering the answers of all participants, the following correlations were found: Regarding the case of the patient suffering from severe and persistent major depressive disorder, the participants’ age, while correcting for time since graduation, was significantly positively correlated to the agreement towards the statement “*In this case, I would not proceed against the patient’s wishes*” (*pr*^*2*^ = 0.20, BCa CI [0.13, 0.28], *p* <.0001). The participants’ time since graduation showed a significant negative correlation with the agreement to this item when corrected for the participants’ age (*pr*^*2*^ = − 0.14, BCa CI [-0.21. -0.07], *p* = 0 0.001). Thus, it revealed a tendency for younger and conversely for participants further from graduation to opt for acting against the patient’s wishes in the specific situation of the depicted case vignette.

### Importance of a patient retaining decision-making autonomy

**Fig. 1:**
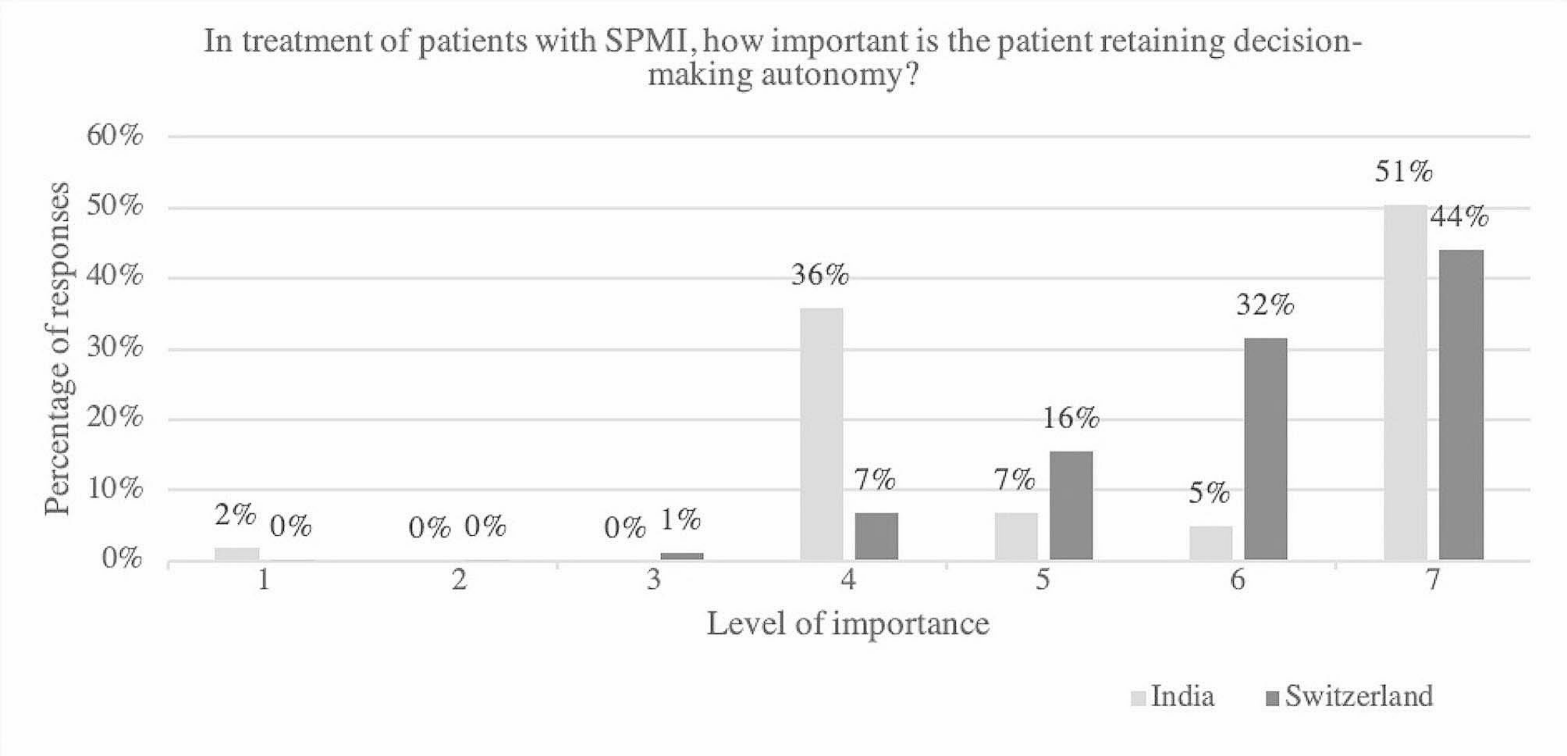
Rating of the statement “In the treatment of patients with SPMI, how important is the patient retaining decision- making autonomy?” comparing answers of the psychiatrists in India and Switzerland; y-axis: percentage of respondents; x-axis: Level of importance ranging from not important [1–3], moderately important [4] to very important [5–7], *N* = 660, with n(India) = 206 and n(Switzerland) = 454

Participants in India answered significantly different than those in Switzerland regarding *autonomous decision making* (*U* = 52254.00, *p* =.01, *r* =.10). The median of the answers given by psychiatrists in India (*Mdn* = 7) was higher than the one of those in Switzerland (*Mdn* = 6). One can see that a vast majority of the participating psychiatrists in Switzerland (91.0%) stated *autonomous decision making* to be of great importance, while in the sample in India a less prominent majority (62.2%) chose this option (see Fig. 1).

### Acting against the patient’s wishes

**Fig. 2:**
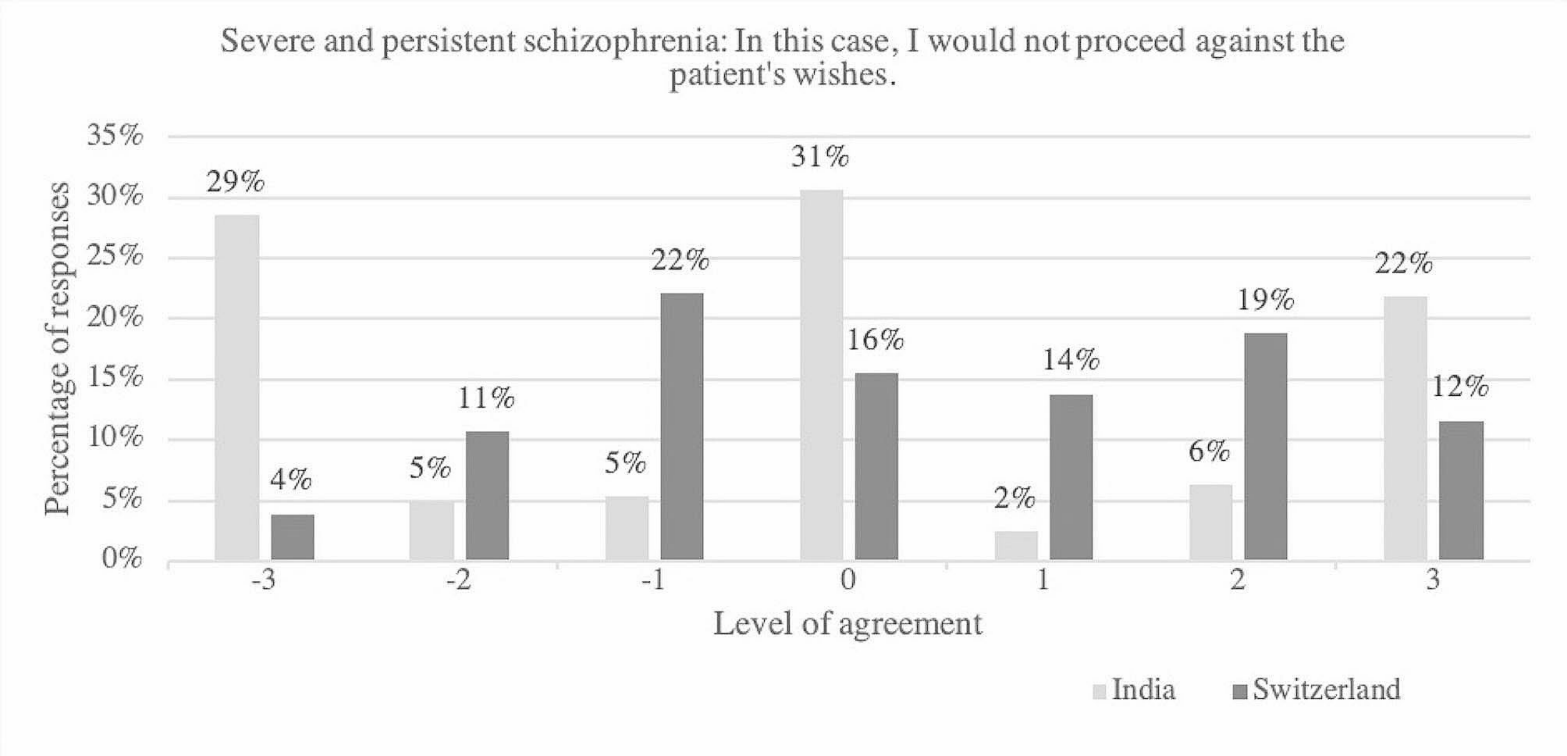
Level of agreement towards the statement “In this case, I would not proceed against the patient’s wishes”, comparing answers of the psychiatrists in India and in Switzerland regarding the case of severe and persistent schizophrenia; y-axis: percentage of respondents; x-axis: level of agreement ranging from disagreement (-3, -2, -1), neutral (0) to agreement [1–3], *N* = 647, with n(India) = 206 and n(Switzerland) = 441

Comparing the answers of participants in India to those of participants in Switzerland to the item *“In this case, I would not proceed against the patient’s wishes”* showed a significant difference regarding both case vignettes (with *U* = 51287.00, *p* =.007, *r* =.11 regarding the case of severe and persistent schizophrenia; and with *U* = 61720.00, *p* <.0001, *r* =.28 regarding the case of severe and persistent major depressive disorder). Considering the case of severe and persistent schizophrenia, the median regarding this item was the same in both groups (*Mdn* = 4). Shown in percentages, 30.5% of the psychiatrist in India compared to 44.2% of those in Switzerland (44.2%) stated to agree on respecting the patient’s wishes with severe and persistent schizophrenia. (see Fig. 2)

**Fig. 3:**
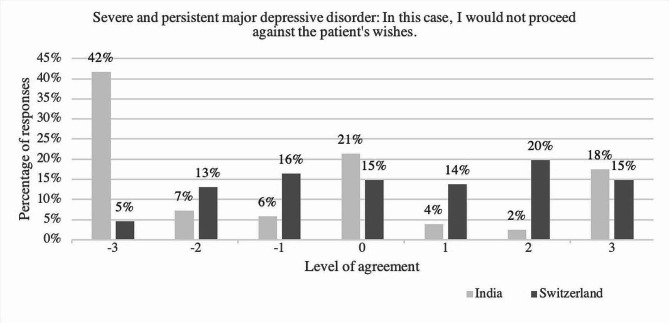
Level of agreement towards the statement “In this case, I would not proceed against the patient’s wishes”, comparing answers of the psychiatrists in India and in Switzerland regarding the case of severe and persistent major depressive disorder; y-axis: percentage of respondents; x-axis: level of agreement ranging from disagreement (-3, -2, -1), neutral (0) to agreement [1–3], *N* = 651, with n(India) = 206 and n(Switzerland) = 445

Concerning the item about respecting the patient’s wishes regarding the case of major depressive disorder, the median (*Mdn* = 3) in the India-group was lower than the median (*Mdn* = 4) in the Switzerland-group. Among the sample in India more than half stated that they would act against the patient’s wishes, while 34.1% of participants in Switzerland chose this option. (see Fig. 3)

### Accepting a temporary decrease in quality of life because of coercive measures

**Fig. 4:**
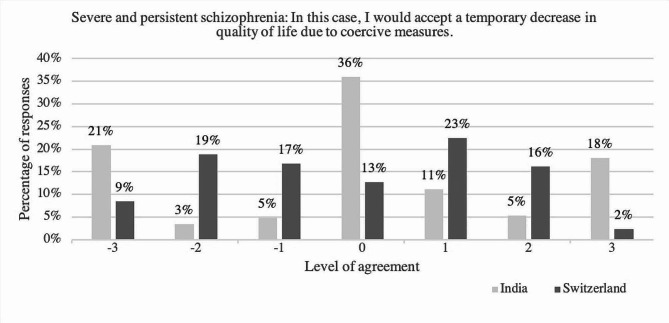
Level of agreement towards the statement “In this case, I would accept a temporary decrease in quality of life as a consequence of coercive measures” comparing answers of the psychiatrists in India and in Switzerland regarding the case of severe and persistent schizophrenia; y-axis: percentage of respondents; x-axis: level of agreement ranging from disagreement (-3, -2, -1), neutral (0) to agreement [1–3], *N* = 653, with n(India) = 205 and n(Switzerland) = 448

Comparing answers to the item “In this case, I would accept a temporary decrease in quality of life due to coercive measures” regarding the case of severe and persistent schizophrenia, showed no significant difference between answers of participants in India and Switzerland (*U* = 43761.50, *p* =.328, *r* =.04) with the same median in both groups (*Mdn* = 4). Not accepting a decrease in quality of life was more frequently chosen by psychiatrists in Switzerland (47%) than by psychiatrists in India (26.7%). (see Fig. 4)

**Fig. 5:**
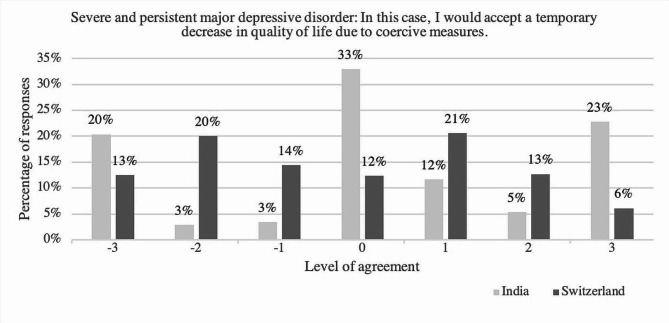
Level of agreement towards the statement “In this case, I would accept a temporary decrease in quality of life as a consequence of coercive measures” comparing answers of the psychiatrists in India and in Switzerland regarding the case of severe and persistent major depressive disorder; y-axis: percentage of respondents; x-axis: level of agreement ranging from disagreement (-3, -2, -1), neutral (0) to agreement [1–3], *N* = 656, with n(India) = 205 and n(Switzerland) = 451

Comparing the answers of participants in India to those of participants in Switzerland regarding the item “In this case, I would accept a temporary decrease in quality of life due to coercive measures” applied to the case of severe and persistent major depressive disorder showed a significant difference (U = 40337.00, *p* =.008, *r* =.10). Regarding the case of major depressive disorder, the equal median in both groups (*Mdn* = 4) support the relative equality in answers of the two groups. Nevertheless, regarding the summarized percentages, participants in Switzerland more frequently stated to disagree on accepting a decrease in life-quality due to coercive measures (44.1%) than their colleagues in India (29.2%). **(**see Fig. 5) Regarding the item “In this case, I would accept a temporary decrease in quality of life as a consequence of coercive measures.” concerning both case vignettes, one participant in the Indian sample did not provide an answer.

### Response style bias between the India and the Switzerland group

Looking at the results across all items of the questionnaire, a tendency for participants in India to choose answers at the end or the middle of the Likert scale, thus options 1, 4 or 7 for the general questions and − 3, 0 or 3 for the case vignettes, was observed. The statistical analysis carried out over all 23 items revealed respondents in the sample in India choosing the option 1, 4, 7 on average more often (*M* = 18.89, *SE* = 0.41) than those in the sample in Switzerland (*M* = 9.29, *SE* = 0.22). This difference, 9.60, *BCa 95% CI* [8.66, 10.48], was significant, *t (324.25)* = 20.70, *p* =.000, with a strong effect of *d = 2.07.*

## Discussion

Most participants in India as well as in Switzerland regarded *autonomous decision making* of patients with SPMI as very important. Nevertheless, answers between the two samples differed significantly with a considerable number of participants in India rating *autonomous decision making* as moderately important while participants in Switzerland opted more uniformly for answers indicating high importance. Regarding the case of severe and persistent major depressive disorder, answers to the item *“In this case, I would accept a temporary decrease in quality of life due to coercive measures”* showed no significant difference between the two samples. Regarding the other items, thus *“In this case, I would accept a temporary decrease in quality of life due to coercive measures”* with respect to the patient with severe and persistent schizophrenia and the item *“In this case, I would not proceed against the patient’s wishes”* regarding both vignettes, answers between the two samples differed significantly. Observed differences suggest Indian psychiatrists being more likely to act against the patient’s wishes or to accept a temporary decrease in quality of life due to coercion compared to Swiss psychiatrists. However, for all results, effect sizes were small.

These findings might reflect the skepticism towards an increased focus on individual autonomy in the Indian context of predominant collectivism and familial interdependence [28, 30, 31]. Besides the individual opinion of the participating Indian psychiatrists, a public opinion critical of individual autonomy might have had a strong influence on the findings. Evidence suggests that individuals in collectivist societies are more likely to conform to community opinion compared to individuals in individualist societies [44].

One reason for the skepticism towards more individualism some Indian people express is based on the fear that more laws emphasizing individual rights might jeopardize trust in families [26, 30] who are contributing to a large part to India’s mental health care [26, 27, 30, 45]. Reducing their decision-making power on the treatment process of a relative suffering from mental illness might diminish their willingness to help [30]. Without the support of families, the capacity of the health care system would be more easily exhausted denying many people access to mental health care [26, 27, 30]. Excluding families from the treatment process might lead to further difficulties as they play a major role in the patient’s life, might influence the development of psychopathological symptoms, and could present an important resource for recovery. Lepping and Raveesh promote the inclusion of the social context when treating mental illness instead of focusing too much on the ethical principle of autonomy [31].

Amendments pursuing the MHCA2017 involve setting up Mental Health Review Boards to control involuntary admissions or coercive measures taken, discussing the possibility of advance directives and the involvement of nominated representatives with patients [46]. The need for more staff to cover the increasing administrative workload contrasts with the lack of financial resources India spends on mental health. Alongside with not taking into account India’s community healthcare and support provided by families, the MHCA2017 projects striven for seem to be detached from India’s economic background [46].

Another downside that comes along with the implementation of the MHCA2017 and more laws and supervisory measures in general is the previously mentioned increasing administrative burden on physicians. They might need to write more detailed documentations of the therapeutic process in anticipation of a potential litigation. This would curtail valuable time which could be better used to build a trustful therapeutic relationship with the patient [17]. A good therapeutic relationship might be further compromised by emerging mistrust due to the possibility of legal action against the physician. Due to the mentioned difficulties in the implementation of the MHCA2017, Indian psychiatrists might be negatively biased against laws fostering individual autonomy in mental health care in general.

Forming and upholding a good therapeutic relationship with the patient is a crucial factor for positive outcomes in psychiatric practice [16]. In 2012, Theodoridou et al. conducted a study to investigate the connection between the quality of the therapeutic relationship and perceived coercion by the patient. The following correlation between the two parameters was detected through structured interviews by applying two standardized scales: higher perceived coercion was associated with a worse therapeutic relationship [15]. In the context of coercive measures, it is thus particularly important to focus on methods to reduce harm to the beneficial therapeutic connection. For example, Shah and Basu proposed a thorough assessment of decision-making capacity, professional communication including repeated disclosure of information to the patient and paying special attention to the patient’s belief about the procedural justice with which the coercive measure has been implemented [29]. To meet such demands, amendments to the mental health care environment need to be designed in a way that allows enough time for the interaction with patients.

Another reason why autonomous decision making might be attributed limited importance might be due to its easy confusion with making decisions independently from others. Autonomy can be translated to self-governance, which is the opposite to being governed by someone else, thus heteronomy. Moreover, autonomous behavior is described as being experienced as willingly enacted and in accordance with one’s own values and beliefs [47]. On the other hand, independence stands for not relying on someone else for support or guidance. Whereby the discussion about where dependence begins and whether certain people lead a completely independent life would go beyond the scope of this work, absolute independence must be a difficult, and above all sad, undertaking. What can be said, however, is that someone can decide completely autonomously to rely on another person for guidance or support and thus to depend on someone else [48]. If autonomous decision making awakens the idea of being left alone to decide, it acquires a negative connotation. The concept of relational autonomy focuses on the interconnectedness of human beings and states that our social surroundings strongly influence identity formation and individual development [49]. It proposes to consider values and beliefs of people close to the patient in a decision-making process characterized by empathy [50]. This concept might be especially well suited for tightly knit collectivist societies, since autonomy is promoted, and social connections are valued at the same time.

What influences might be responsible for the tendency of Swiss participants to rate autonomous decision making higher than Indian participants? In Hofstede’s Individualism-Collectivism Scale, Western countries like Switzerland achieve high scores of Individualism [22] which might explain a strong focus on respecting patient’s autonomy among Swiss psychiatrists. Psychiatrists in Switzerland might also be more hesitant to use coercion, due to the fear of a possible litigation by their patients or relatives.

Moreover, European psychiatry has a history of overriding the patient’s autonomy [31]. Moreover, in both countries psychiatry itself substantially changed over time and was influenced by different key concepts and figures [14, 21, 51–55]. While elaborating differences in psychiatric history among India and Switzerland would go beyond the scope of this work, it is essential to note that psychiatry is highly intertwined with the prevailing society, culture, and politics.

### Strengths and limitations

The low response rate in India is a limitation of our study because of a potential pre-selection bias for psychiatrists with a particular interest in the topic, reducing generalizability of the findings. Moreover, data collection at different times, with an interval of more than four years, poses a limitation to the study, as the attitudes on coercive measures might also have evolved in the meantime. In both groups a similar gender distribution was found. Nevertheless, female psychiatrists might be over-represented in the Indian sample as a study conducted in 2009 estimated only 15% of psychiatrists in India being female [56] compared to a rather realistic gender distribution in the Swiss sample (for gender-distribution among psychiatrist in Switzerland see [57]). In terms of the participants’ age and work experience, the sample in India consisted of significantly younger and closer to graduation psychiatrists than the sample in Switzerland, which might reduce the comparability of the results. This might especially affect answers to the item *“In this case, I would not proceed against the patient’s wishes”* regarding a case of a patient with severe and persistent major depressive disorder due to the significant correlations found: In both samples, younger participants would rather proceed against the patient’s wishes in the depicted case. The on average younger age of the participants in the Indian sample might therefore present a possible confounding factor. In contrast, when looking at the time since graduation, the following correlation was found regarding the same item: participants who graduated more recently were more likely to act according to the patient’s wishes. One possible explanation could be as follows: younger psychiatrists more often work in inpatient acute wards where coercive measures are used more frequently than for example in outpatient mental health care [58]. Being frequently confronted with coercive measures might lead to a lower-threshold application of these. Conversely, participants who recently graduated from medical school might remember lectures on medical ethics more easily which might be the reason for them to be more cautious when it comes to the use of coercion.

In the present study, coercive measures were defined as a broad concept, and participants were neither provided with a definition of coercion nor a list of measures understood as being coercive. The item “In the treatment of patients with SPMI, how important is the patient retaining decision making autonomy?” was aimed to explore the importance given to the avoidance of coercion, and therefore, preserving the patients’ autonomy generally. The first item regarding the case vignettes was aimed to assess whether coercion of any kind would be applied in specific situations. The last item focused on the extent of coercion applied expressed by a temporary reduction in the patient’s quality of life due to the coercive measure. The study focused on the importance of patients’ autonomy, application of any kind of coercion in a specific situation, and the degree of coercion applied rather than comparing the opinions on specific coercive methods. On the one hand, this is seen as a strength of the study as it allows to explore the participants individual conception of coercion. On the other hand, it makes the answers less comparable with each other as everyone might have different types of coercion in mind while answering the questions. It would be interesting to explore differences in attitudes on more specific coercive measures in future research. It should be added that participants were not provided by a uniform definition of autonomous decision-making which might have reduced the comparability of the results. Moreover, the questionnaire’s adaptations and its translation to English might have led to a reduced comparability of the Indian to the Swiss answers. Further, response style bias, representing different answering tendencies across cultures regardless of the question’s content, pose a further difficulty when performing cross-cultural research [59]. The analysis across all items of the questionnaire showed participants in India choosing answers at the end or the middle of the Likert scale significantly more often than those in Switzerland. This different response behavior between the two groups might present a considerable confounder when comparing the results. Yet, summarizing the 7-point Likert scale into three categories might have had a balancing effect on the found response style bias. This is supported by the fact that the same statistical analyses using a collapsed Likert scale, thus with only three response options, produced qualitatively the same results (see supplementary materials). Another limitation arises from the huge difference in geographical size and number of inhabitants of the two compared countries, as attitudes might also differ regarding the region of origin within the same country. This national variability might be more significant in a larger country like India.

To close, it should be considered that the survey-items analyzed for this paper only allow a rough assessment of the participants’ opinions and cannot deal conclusively with this very complex topic. Choosing answers in a survey cannot be compared with taking decisions on coercive measures in real life, burdened with the pressure to make a quick yet considered decision facing the imminent harm to self or others. Further research is required to support our findings and gain more insight in cultural differences on the attitude towards autonomous decision making. It might be especially interesting to explore personal attitudes by a more qualitative methodology, for example by a different questionnaire design including assorting different aspects in the treatment of patients with SPMI according to their importance. Also, it might be interesting to present case vignettes with situations that could possibly lead to coercive measures followed by interviewing psychiatrists on their attitudes and arguments for decision making.

## Conclusions

The present study helps to get a general impression of differences in attitudes towards coercive measures depending on whether Indian or Swiss psychiatrists were interviewed. It revealed a tendency of Indian participants to value autonomous decision making of patients suffering from SPMI as slightly less important than their Swiss colleagues. On average, interviewed psychiatrists in the Indian sample would rather act against the patient’s wish than psychiatrists in the Swiss sample. A temporary decrease in quality of life due to coercive measures in a patient suffering from major depressive disorder met greater acceptance among surveyed Indian than Swiss psychiatrists. However, for all findings effect sizes were small and the implications of the results should therefore not be overestimated.

In difficult situations that arise in everyday clinical practice including when it comes to coercive measures, it is not a matter of evaluating autonomy alone, but of weighing it against other values. Moreover, it might be worthwhile to examine palliative care approaches with a focus on quality of life and reduction of suffering to provide patients with SPMI with the best possible care and reduce coercion.

### Electronic supplementary material

Below is the link to the electronic supplementary material.

Supplementary Material 1

## Data Availability

The datasets used and/or analyzed during the current study are available from the corresponding author on reasonable request.

## Abbreviations

SPMI: Severe and persistent mental illness
MHCA2017: Mental Health Care Act 2017

## References

[1] Ruggeri M, Leese M, Thornicroft G, Bisoffi G, Tansella M (2000). Definition and prevalence of severe and persistent mental illness. Br J Psychiatry.

[2] Zumstein N, Riese F (2020). Defining severe and persistent Mental Illness-A pragmatic utility Concept Analysis. Front Psychiatry.

[3] Hotzy F, Moetteli S, Theodoridou A, Schneeberger AR, Seifritz E, Hoff P (2018). Clinical course and prevalence of coercive measures: an observational study among involuntarily hospitalised psychiatric patients. Swiss Med Wkly.

[4] Wissenschaften SAdM. Zwangsmassnahmen in Der Medizin. Schweizerische Akademie der Medizinischen Wissenschaften SAMW; 2015. [.

[5] Di Francesco R, Drohé L, Kronenberg P, ANQ. Nationaler Verein für Qualitätsentwicklung in Spitälern und Kliniken, Bern; w hoch 2 GmbH, Bern (2023). Symptombelastung und Freiheitsbeschränkende Massnahmen stationäre Psychiatrie Erwachsene. Nationaler Vergleichsbericht 2022. 2023.

[6] Schuler D, Tuch A, Peter C. Psychische Gesundheit in Der Schweiz. Monitoring 2020. Schweizerisches Gesundheitsobservatorium (Obsan); 2020.

[7] Raboch J, Kalisová L, Nawka A, Kitzlerová E, Onchev G, Karastergiou A (2010). Use of coercive measures during involuntary hospitalization: findings from ten European countries. Psychiatr Serv.

[8] Gowda GS, Lepping P, Ray S, Noorthoorn E, Nanjegowda RB, Kumar CN (2019). Clinician attitude and perspective on the use of coercive measures in clinical practice from tertiary care mental health establishment - A cross-sectional study. Indian J Psychiatry.

[9] Srinivasan TN, Thara R (2002). At issue: management of medication noncompliance in schizophrenia by families in India. Schizophr Bull.

[10] Hoff P. Autonomie, ein zentraler, aber sperriger Begriff der Psychiatrie. Swiss Archives of Neurology, Psychiatry and Psychotherapy2017. pp. 175– 82.

[11] Berlin I (2017). Two concepts of liberty.

[12] Hick C (2007). Medizinethisches Argumentieren.

[13] Schwartz B (2000). Self-Determination. The tyranny of Freedom.

[14] Kant I. Grundlegung zur metaphysik der sitten: L. Heimann; 1870.

[15] Theodoridou A, Schlatter F, Ajdacic V, Rössler W, Jäger M (2012). Therapeutic relationship in the context of perceived coercion in a psychiatric population. Psychiatry Res.

[16] Hoff P (2019). Compulsory interventions are challenging the identity of Psychiatry. Front Psychiatry.

[17] Harbishettar V, Krishna KR, Srinivasa P, Gowda M (2019). The enigma of doctor-patient relationship. Indian J Psychiatry.

[18] Pollmächer T (2019). [Autonomy focusing as guiding idea of minimally restrictive psychiatry]. Nervenarzt.

[19] Hürlimann D, Trachsel M. die fürsorgerische Unterbringung von Urteilsfähigen zulässig? SWISS MEDICAL FORUM2017.

[20] Beauchamp TL, Childress JF. Principles of Biomedical Ethics. 7 ed. Oxford University Press; 2009.

[21] Ziegler A. Zwangsunterbringung Und Zwangsbehandlung. Klinische Ethik. Springer Medizin; 2007. pp. 170–83.

[22] Hofstede G. Dimensionalizing cultures: the Hofstede Model in Context. Online readings in psychology and culture. International Association for Cross-Cultural Psychology; 2011.

[23] Wong YJ, Wang S-Y, Klann EM (2018). The Emperor with no clothes: a critique of Collectivism and Individualism.

[24] Khandelwal SK, Jhingan HP, Ramesh S, Gupta RK, Srivastava VK (2004). India mental health country profile. Int Rev Psychiatry.

[25] Kala AK (2012). Covert medication; the last option: a case for taking it out of the closet and using it selectively. Indian J Psychiatry.

[26] Gowda GS, Enara A, Raveesh BN, Gowda M (2019). Is it the right time to implement Community Treatment Order in India?. Indian J Psychiatry.

[27] Trivedi JK (2001). Implication of erwadi tragedy on mental health care system in India. Indian J Psychiatry.

[28] Bhola P, Chaturvedi SK. Through a glass, darkly: ethics of mental health practicioner-patient relationships in traditional societies. Int J Cult Mental Health2017.

[29] Shah R, Basu D (2010). Coercion in psychiatric care: global and Indian perspective. Indian J Psychiatry.

[30] Kala A (2013). Time to face new realities; mental health care bill-2013. Indian J Psychiatry.

[31] Lepping P, Raveesh BN (2014). Overvaluing autonomous decision-making. Br J Psychiatry.

[32] Group TWB. World Health Organization Global Health Expenditure database [.

[33] WHO WHO. https://apps.who.int/gho/data/view.main.HWF11v [.

[34] Raveesh BN, Pathare S, Noorthoorn EO, Gowda GS, Lepping P, Bunders-Aelen JG (2016). Staff and caregiver attitude to coercion in India. Indian J Psychiatry.

[35] Lepping P, Steinert T, Gebhardt RP, Röttgers HR (2004). Attitudes of mental health professionals and lay-people towards involuntary admission and treatment in England and Germany–a questionnaire analysis. Eur Psychiatry.

[36] Molodynski A, Turnpenny L, Rugkåsa J, Burns T, Moussaoui D (2014). Coercion WAoSPIWGo. Coercion and compulsion in mental healthcare-an international perspective. Asian J Psychiatr.

[37] Stoll J, Hodel MA, Riese F, Irwin SA, Hoff P, Biller-Andorno N (2021). Compulsory interventions in severe and persistent Mental illness: a Survey on attitudes among psychiatrists in Switzerland. Front Psychiatry.

[38] Trachsel M, Hodel MA, Irwin SA, Hoff P, Biller-Andorno N, Riese F (2019). Acceptability of palliative care approaches for patients with severe and persistent mental illness: a survey of psychiatrists in Switzerland. BMC Psychiatry.

[39] Stoll J, Mathew A, Venkateswaran C, Prabhakaran A, Westermair AL, Trachsel M. Palliative Psychiatry for patients with severe and persistent Mental illness: a Survey on the attitudes of psychiatrists in India compared to psychiatrists in Switzerland. Frontiers in Psychiatry2022. 10.3389/fpsyt.2022.858699 PMC9178077 35693967

[40] Baweja R, Singareddy R (2013). Concomitant use of maintenance ECT and vagus nerve stimulation for more than 10 years in treatment-resistant depression. Am J Psychiatry.

[41] Brenner HD, Dencker SJ, Goldstein MJ, Hubbard JW, Keegan DL, Kruger G (1990). Defining treatment refractoriness in schizophrenia. Schizophr Bull.

[42] Field A (2013). Discovering statistics using IBM SPSS statistics.

[43] Cohen J (1992). A power primer. Psychol Bull.

[44] Triandis HC. Dialectics between cultural and cross-cultural psychology. Asian Journal of Social Psychology: Blackwell Publishers Ltd with the Asian Association of Social Psychology and the Japanese Group Dynamics Association; 2000. pp. 185– 95.

[45] Avasthi A (2010). Preserve and strengthen family to promote mental health. Indian J Psychiatry.

[46] Math SB, Basavaraju V, Harihara SN, Gowda GS, Manjunatha N, Kumar CN (2019). Mental Healthcare Act 2017 - aspiration to action. Indian J Psychiatry.

[47] Deci EL, Ryan RM. Self-determination theory. In: Van Lange PAM, Kruglanski AW, Higgins ET, editors. Handbook of theories of social psychology: volume. Volume 1. SAGE Publications Ltd; 2012.

[48] Chirkov V, Ryan RM, Kim Y, Kaplan U (2003). Differentiating autonomy from individualism and independence: a self-determination theory perspective on internalization of cultural orientations and well-being. J Pers Soc Psychol.

[49] Dove ES, Kelly SE, Lucivero F, Machirori M, Dheensa S, Prainsack B (2017). Beyond individualism: is there a place for relational autonomy in clinical practice and research?. Clin Ethics.

[50] Heidenreich K, Bremer A, Materstvedt LJ, Tidefelt U, Svantesson M (2018). Relational autonomy in the care of the vulnerable: health care professionals’ reasoning in Moral Case Deliberation (MCD). Med Health Care Philos.

[51] Reil JC. Rhapsodieen über die Anwendung der psychischen Kurmethode auf Geisteszerrüttungen. 1818.

[52] Philippe Pinel PdM-SzP. Philosophisch-medizinische Abhandlung über Geistesverirrungen oder Manie. 1801.

[53] Foot J (2014). Franco Basaglia and the radical psychiatry movement in Italy, 1961-78. Crit Radic Soc Work.

[54] Foucault M. The Birth of the Clinic. 1963.

[55] Bonnie RJ (2002). Political abuse of psychiatry in the Soviet Union and in China: complexities and controversies. J Am Acad Psychiatry Law.

[56] Sood M, Chadda RK (2009). Women in psychiatry: a view from the Indian subcontinent. Indian J Psychiatry.

[57] Georgescu D (2009). Psychiatry in Switzerland. Int Psychiatry.

[58] Morandi S, Silva B, Mendez Rubio M, Bonsack C, Golay P (2021). Mental health professionals’ feelings and attitudes towards coercion. Int J Law Psychiatry.

[59] Clarke I. Extreme Response Style in Cross-Cultural Research: An Empirical Investigation. Journal of Social Behavior and Personality2000. pp. 137– 52.

