# Supplementary material for: Comparing attitudes towards compulsory interventions in severe and persistent mental illness among psychiatrists in India and Switzerland
Source: BMC Psychiatry. 2024 Apr 18;24:295. doi: 10.1186/s12888-024-05710-6 (PMC11025243; doi:10.1186/s12888-024-05710-6)
Supplement: Supplementary file 1 — Supplementary Material 1 [file 12888_2024_5710_MOESM1_ESM.docx]

## Supplementary material

Table 1: Survey Items

|  | |
| --- | --- |
| **I: Questions regarding the treatment of patients with severe and persistent mental illness (SPMI)** | |
|  |  |
| In the treatment of patients with SPMI, how important are the following: | |
|  |  |
|  | a) curing the illness |
|  | b) reduction of suffering |
|  | c) patient’s ability to function in daily life |
|  | **d) patient retaining decision making autonomy** |
|  | e) impeding suicide |
|  |  |
| **II: Case vignettes:** Please provide an evaluation of the case vignettes. | |
|  |  |
|  | **a) In this case, I would not proceed against the patient’s wishes.** |
|  | b) For this patient, any further intervention to cure their schizophrenia/depression would most likely prove futile. |
|  | c) In this case, I would be comfortable with a reduction of life expectancy in order to increase or maintain the patient’s quality of life (if consistent with their goals). |
|  | **d) In this case, I would accept a temporary decrease in quality of life as a consequence of coercive measures.** |
|  | e) I would not be surprised if this patient died within the next 6 months. |
|  |  |

Table 2: Case vignettes

|  | |  |
| --- | --- | --- |
| **Patient 1:**   - 33-year-old male - Schizophrenia with onset at age 17; no significant comorbidities - Positive symptoms: auditory and visual hallucinations, persecutory delusions - Negative symptoms: apathy, social withdrawal, poverty of speech (all rated severe) | | |
|  |  | |
|  | Despite long-lasting high-dose pharmacological treatment (several atypical neuroleptics, haloperidol, clozapine, and their combinations), as well as electroconvulsive therapy, the patient has never been free from positive or negative symptoms. Multiple psychotherapies employing various approaches have also failed to stabilize the patient or to improve his quality of life. He does not wish to continue treatment because he feels it is too intrusive. While the positive symptoms predominated in the years immediately following his initial diagnosis, he developed severe negative symptoms, as well as aggression and self-injurious behavior, including burning himself with cigarettes. The negative symptoms and his strong functional deficits are exacerbated by chronic unemployment and an inability to live independently. Additionally, the patient has no family system, and his persisting illness has left him completely isolated, with no social contacts and no hobbies or interests. Two experts have declared that he possesses decision-making capacity regarding his illness and its treatment. | |
|  |  | |
| **Patient 2:**   - 40-year-old male - Recurrent Major Depressive Disorder; no significant comorbidities - Somatic symptoms: energy loss, insomnia, and fatigue - Persistent suicidal ideation over the past 20 years; current acute and concrete suicidal intent | | |
|  | |  |
|  | The patient underwent different forms of intensive, long-term, evidence-based psychotherapy, including specialized approaches such as cognitive behavioral analysis system of psychotherapy (CBASP) and interpersonal psychotherapy (IPT). Both psychotherapy alone and in combination with adequate treatment trials of antidepressants (selective serotonin reuptake inhibitors, tricyclic antidepressants, venlafaxine, augmentation with lithium and antipsychotic medications (quetiapine and aripiprazole)) failed to improve his depression, and the patient experienced significant adverse effects from several of the medications. Exhausted, he has decided to undergo electroconvulsive therapy as a last resort. However, maintenance electroconvulsive therapy again proved insufficient to prevent the reappearance of suicidal ideation; indeed, his symptoms worsened. Experiencing severe hopelessness, the patient states that his quality of life is very poor, that he doesn’t want to deal with his illness anymore, and that he plans to commit suicide in the near future. Two experts have declared that he possesses decision-making capacity regarding his illness and its treatment. | |
|  | |  |

Table 3 Results from data collapsed in three answering categories: not important/disagreement, moderately important/neutrality and very important/agreement.
